# Trends in the geographic inequality of advanced practice nursing workforce in cancer care in Japan from 1996 to 2022: a panel data analysis

**DOI:** 10.1186/s12960-024-00922-z

**Published:** 2024-05-27

**Authors:** Tomoko Tamaki, Noriko Morioka, Ako Machida, Masayo Kashiwagi

**Affiliations:** 1 Juntendo University Hospital, 3-1-3 Hongo, Bunkyo-ku, Tokyo, 1130033 Japan; 2 Department of Nursing Health Services Research, Graduate School of Health Care Sciences, Tokyo Medical and Dental University, 1-5-45 Yushima, Bunkyo-ku, Tokyo, 1138510 Japan

**Keywords:** Cancer care, Nurse workforce, Advanced practice nurse, Geographic distribution, Ecological study

## Abstract

**Background:**

Cancer was ranked as the second leading cause of global mortality in 2019, with an increasing incidence. An adequate workforce of healthcare professionals with special skills and knowledge in cancer care is vital for addressing the disparities in cancer prognosis. This study aimed to elucidate the trends in the advanced practice nursing workforce (APNW) in cancer care, which included certified nurse specialists (CNSs) and certified nurses (CNs) in each prefecture of Japan from the system's inception to the present. Further, it sought to analyze the regional disparities and compare these trends with other healthcare resources to identify contributing factors associated with the APNW in cancer care in each prefecture.

**Methods:**

We performed a panel data analysis using publicly available data on the APNW in cancer care in each prefecture of Japan from 1996 to 2022. Gini coefficients were calculated to examine the trends in geographic equality. Univariate and multivariable fixed effect panel data regression models were used to examine regional factors associated with an APNW in cancer care.

**Results:**

From 1996 to 2012, the APNW in cancer care increased from four to 6982 staff, while their Gini coefficients decreased from 0.79 to 0.43. However, from 2012 to 2022, the Gini coefficients decreased slightly from 0.43 to 0.41. The coefficient value was comparable to that for the disparity between hospital doctors (0.43) but more pronounced compared to those for other medical resources, such as hospitals (0.34), hospital nurses (0.37), and designated cancer care hospitals (0.29). The APNW in cancer care in each prefecture was significantly associated with a higher number of designed cancer care hospitals in the previous year (see first quartile, the coefficient for second quartile: 0.31, 95% confidence interval (CI) 0.21–0.40), and a fewer number of hospital doctors (− 1.89, 95%CI − 2.70 to − 1.09).

**Conclusions:**

The size of the APNW in cancer care has increased since the system was established in 1996 up till 2022. With the increase in numbers, geographic inequality narrowed until 2012 and has since then remained stagnant.

**Supplementary Information:**

The online version contains supplementary material available at 10.1186/s12960-024-00922-z.

## Background

According to the Global Burden of Disease, cancer was the second leading cause of death worldwide in 2019, with a 26% increase over the past 10 years [1, 2]. Ensuring an adequate workforce in cancer care is crucial for addressing the disparities in cancer prognosis [3]. The presence of nursing teams with specialised knowledge and skills in cancer care has been shown to improve overall cancer survival rates significantly [3, 4]. In particular, the advanced practice nursing workforce (APNW), which includes oncology clinical nurse specialists, plays various vital roles not only in delivering direct patient care [5–7] but also in the quality management of the entire nursing team, such as education and leadership [8–10]. Despite the contributions of the advanced practice nurses, the geographic inequality between the APNW and the general nurses has been noted [5, 9]. In Japan, cancer is also an important social issue and was the leading cause of death in 2022, claiming the lives of approximately 380,000 individuals and accounting for 24.6% of all deaths nationwide [11]. With the increase in cancer patients, the demand for standardisation in cancer treatment has grown in Japan. An APNW provides qualitative care in cancer treatment, and their distribution is crucial for standardising cancer care. In 1996, the Japanese Nursing Association introduced certified nurse specialists (CNSs) and certified nurses (CNs) as categories within the APNW. These workers comprise over 6900 advanced practice nursing workers in cancer care [12, 13]. They engage in medication adjustments to manage symptoms, directly intervene and assess complex patient backgrounds, and handle nurse consultations [14, 15]. As per the policy of cancer equalisation, the provision of quality nursing care by the APNW in cancer care should also be distributed equally geographically. There are reports on the association between the hospital nurse workforce in the sub-national or the medical area level and medical reimbursement, socioeconomic conditions, population, population density, average income per capita, number of nursing school graduates, previous year’s wages for nurses per hour, the proportion of the population aged 65 and older, and the regional divisions [16–18]. However, no nationwide survey on the distribution of the APNW in cancer care in Japan has been conducted, and research on the nursing workforce for cancer care is limited, with only one study based on research from the UK conducted in 1996, highlighting the scarcity of global data on cancer nurses [3].

This study aims to:

1. Elucidate the trends in the APNW in cancer care in each prefecture in Japan from the inception of the system to the present, including regional disparities.
2. Identify the contributing factors associated with the number of APNW in cancer care in each prefecture.

### Background of policy and advanced practice nursing system in cancer care in Japan

In 2000, the Japanese government initiated a nationwide cancer policy to achieve equitable cancer care. In 2001, designated cancer hospitals were established, with one in each prefecture serving as the core hospital for cancer care. In 2007, the Cancer Control Act was enforced, targeting the promotion of cancer prevention, early detection, equalisation of cancer care, and the advancement of cancer research [19].

Before the government-led promotion of policy for cancer care, the Japanese Nursing Association (JNA) introduced the certification system for APNW in cancer care in 1996 within the framework of the certification system for APNW. In Japan, the certification system for APNW was introduced in 1996 by the JNA [20]. Although, globally, advanced practice nurses require minimum master’s degree [21], JNA introduced CN requires 600 h or more additional training and five years or more of clinical experience but does not require master’s degree and CNS requires master’s degree and five years or more of clinical experience as providers of advance practice nursing, because only eight universities or colleges opens for master’s course in nursing as of 1996, and there was still a small proportion of nurses with a bachelor in nursing. The CNs are involved in practice, guidance, and consultation, must have at least three years of practical experience, and must undergo six months of training at an educational institution without the requirement of a master’s degree [20]. Although the expected roles are different for CNS and CN: CNS with six roles (practice, consultation with care providers, coordination between health and social care professionals, ethical coordination, education, and research), and CN with four roles (high standards of nursing practice, instruction, and consultation with nursing professionals and others, with a high level of clinical reasoning and judgement of pathological conditions.), both CNSs and CNs deliver advanced practice nursing in Japan [22, 23]. Since 2020, the JNA has been reforming the CNS and CN systems by consolidating fields (See Appendix 1).

Regarding APNW in cancer care, there are two categories of CNSs (cancer nursing and radiation nursing) and five categories of CNs (palliative care, breast cancer nursing, radiation oncology nursing, and cancer chemotherapy and immunotherapy). The Cancer Control Act and related notice by the Director-General of the Health Services Bureau, Ministry of Health, Labor and Welfare [19] requires designated cancer care hospitals to have at least one CNS or CN in cancer care. Further, it recommends that staff include at least one oncology CNS or CN in each radiology and chemotherapy department [24]. Furthermore, financial incentives in the fee schedule are provided to the APNW in cancer care for their participation in specialised teams and placement in hospitals [25].

## Methods

### Study design and data source

We conducted a panel data analysis using publicly available data on the APNW in cancer care from 47 prefectures (a prefecture is a subnational unit in Japan) from the year of certification commencement in 1996 to 2022. JNA aggregated the data by prefecture based on the address of the CNS or CN’s place of employment, which is registered to the JNA certification system.

### Variables

#### Dependent variable

The dependent variable was the number of advanced nurses in cancer care. This category includes cancer nursing CNSs, radiation nursing CNSs, palliative cancer care CNs, breast cancer CNs, radiation oncology CNs, cancer chemotherapy CNs, cancer chemotherapy and immunotherapy nursing CNs. Data on the headcount numbers of CNSs and CNs were obtained from the JNA [12, 13].

#### Characteristics of the prefectures

As prefecture variables, we used the nurse supply, medical care supply, and socioeconomic status.

##### Nurse supply

Data on the hourly wage of nurses and the number of hospital nurses were used for nurse supply variables. Data on nurses’ hourly wages were collected from the Basic Survey on Wage Structure [26]. This variable examines the impact of nurses’ wages on the number of nurses. Data on the number of nurses in hospitals were extracted from the Medical Facility Survey [27].

##### Medical care supply

We used the number of hospital doctors and designed cancer care hospitals as the medical care supply variables. Data on the number of hospital doctors were retrieved from Medical Facility Surveys [27] and hospital reports [28]. Data on the number of designated cancer care hospitals from 2001 to 2022 were collected from the list of designated cancer care hospitals [29–32], materials from the study group on the designation of base hospitals for cancer treatment coordination [33], and in-hospital cancer registries [34]. This latter variable was used to determine whether the presence or absence of a cancer-care hospital affected the number of nurses. Designated cancer care hospitals were defined as prefectural cancer care base hospitals, regional cancer care base hospitals, specific-area cancer care collaboration hospitals, regional cancer care hospitals, paediatric cancer care base hospitals, and central organisations for paediatric cancer.

##### Socioeconomic status

We used the variables population, percentage of the population aged 65 years and older, and average income per capita, and the number of cancer care for the socioeconomic status of prefectures.

The total population data for each prefecture was collected and calculated from the Vital Statistics and National Population Census based on the Basic Resident Registers [35]. The proportion of the population aged 65 and older for each prefecture was obtained from demographic and household surveys [35]. The average income per capita data for the previous year were collected from the Cabinet Office's Prefectural Accounts [36]. The number of cancer care was only available from 2016 to 2019 obtained from the Population-based cancer registry [37]. Due to data availability limitations, the number of cancer patients was only used in the sensitivity analysis.

### Analysis

We analysed the means, quartiles, and range of characteristics of prefectural variables for every five years. To present the trends in the APNW and its distribution per 10,000 population, we used bar charts and box-and-whisker plots, respectively. To assess the trends in the degree of equality between the APNW and other medical resources per population, we calculated the Gini coefficient for the APNW in cancer care, hospital doctors, hospital nurses, and the designated cancer care hospital population. The Gini coefficient is the ratio of the area between the Lorenz curve and the equality line to the total area under the equality line. The index has a value between zero and one, with zero representing perfect equality and one representing perfect inequality.

The Gini coefficient was calculated using the following equation [38]:

$$G=1-\sum_{i=0}^{n}\left({Y}_{i+1}+{Y}_{i}\right)\times ({X}_{i+1}+{X}_{i})$$

*n*: Total number of prefectures.

*Y*_*i*_: Cumulative percentages of APNW in cancer care (and other medical resources) in the prefecture.

*X*_*i*_: Cumulative percentage of the population belonging to the prefecture.

As a sensitivity analysis, we calculated the Gini coefficients for the number of cancer patients the instead of the total population only data available from 2016 to 2019.

To examine the factors associated with the APNW in cancer care at the prefectural level, a panel dataset was created, with each prefecture as the unit of analysis. Univariate and multivariable panel data regression models with a fixed effects approach were used. The dependent variable was the APNW in cancer care in each prefecture. From the literature review [17, 18], we selected the population in the previous year, the percentage of the population aged + 65 in the previous year, the average income per capita in the previous year, the number of hospital nurses in the previous year, nurse’s hourly wages in the previous year, the number of hospital doctors in the previous year, and the number of designated cancer care hospitals in the previous year, as time-varying independent variables. The numbers of the APNW in cancer care, hospital doctors, hospital nurses, and the average income per capita were log-transformed because the distribution was skewed to the right. Nurses’ wages were also log-transformed to examine the wage elasticity of the nursing labour supply as follows.

$$\begin{aligned} \log \,({\text{number of APNW in cancer care}})_{{i{\text{y}}}} = \,& \beta _{0} + \beta _{1} \left( {{\text{year}}} \right) \\ & + \beta _{{2 - 4}} \left( {{\text{dummy of the qurtiles of the total population}}} \right)_{{i\left( {{\text{y}} - 1} \right)}} \\ & + \beta _{5} \left( {{\text{percentage of the population aged}} + 65} \right)_{{i\left( {{\text{y}} - 1} \right)}} \\ & + \beta _{6} {\text{log}}\left( {{\text{number of hospital nurse}}} \right)_{{i\left( {{\text{y}} - 1} \right)}} \\ & + \beta _{7} \left( {{\text{average income per capita}}} \right)_{{i\left( {{\text{y}} - 1} \right)}} \\ & + \beta _{8} \left( {{\text{average hourly salary of nurse}}} \right)_{{i\left( {{\text{y}} - 1} \right)}} \\ & + \beta _{{9 - 11}} \left( {{\text{dummy of the qurtiles the number of designated cancer care hospitals}}} \right)_{{i\left( {{\text{y}} - 1} \right)}} \\ & + u_{i} \\ \end{aligned}$$

where,

*i* = prefecture.

y = year.

${u}_{i}=$ prefecture-specific intercept.

Multicollinearity was assessed by correlations among independent variables < 0.7 in each year. The number of hospitals, hospital nurses, and hospital doctors highly correlated (Pearson’s *r* > 0.90); we created three models: model 1 with hospital nurses (above equation), model 2 with hospital doctors instead of hospital nurses, and model 3 with number of hospitals. The fittingness of models was assessed by the overall R-squared. Descriptive statistics, Gini coefficients, and regression analyses were performed using the panel data. Stata (version 16.1) was used for the analyses, with a significance level of 5%.

### Ethical consideration

No individual data points were utilised in this study. All data were obtained from open sources and are available on the website. This study adhered to the principles of the Declaration of Helsinki.

## Results

**Fig. 1:**
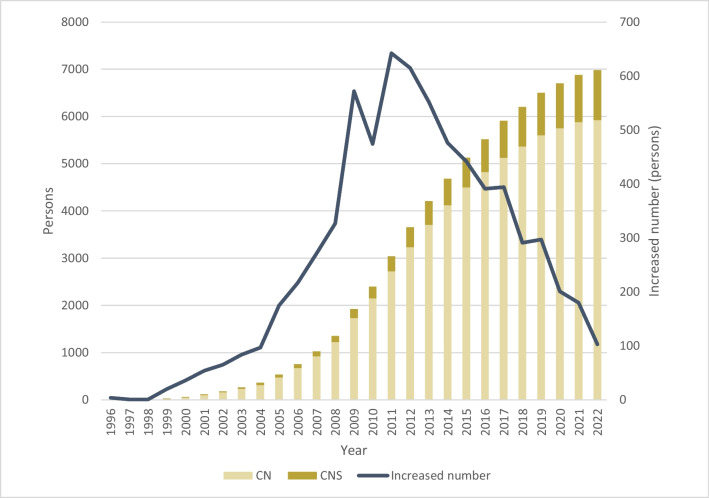
Trends in the number of advanced practice nursing workers in cancer care from 1996 to 2022. CN: Certified nurse, CNS: Certified nurse specialist

Figure 1 shows the changes in the APNW in cancer care in Japan over time. The APNW in cancer care increased from four persons in 1996 to 6982 in 2022. The number of people attended to by the APNW in cancer care increased from four in 1996 to 642 in 2011, followed by a marked decline to 103 in 2022.

Table 1 and Appendix 2 show the characteristics of each prefecture and the changes in various variables from 1996 to 2022. While the medians of the total population decreased from 1795 thousand persons to 1605 thousand persons, the percentage of the population aged 65 years and older increased from 18 to 31%, and the number of nurses per 10,000 population increased from 40 to 70. However, the wages (approximately US$13.3/h as of 25 October 2023) have not changed significantly over the last 26 years.

**Table 1:** Characteristics of prefectural variables (Prefectures: *N* = 47)

	1996	2001	2006	2011	2016	2022
Number of hospital nurses per 10,000 people [27, 35]						
Min	20.0	38.9	28.3	35.1	41.8	−
25%tile	32.1	53.7	44.1	51.6	58.8	−
50%tile	39.5	66.9	51.4	62.2	70.3	−
75%tile	46.7	77.7	60	71.5	81.2	−
Max	52.9	104.4	74.9	90	105.2	−
Nurses’ wages, 1000yen/h [26]						
Mean	−	2.30	2.21	2.27	2.33	−
SD	−	0.19	0.18	0.2	0.16	−
Number of doctors per 10,000 people [27, 28, 35]						
Min	8.8	9.2	9.7	11.0	12.1	−
25%tile	11.2	11.8	12.6	13.8	15.0	−
50%tile	12.8	13.6	14.1	15.9	17.0	−
75%tile	14.6	15.5	16.5	18.2	19.5	−
Max	18.2	19.7	20.9	21.9	24.6	−
Number of hospitals per 10,000 people [28, 35]						
Min	0.871	0.846	0.824	0.804	0.790	−
25%tile	0.335	0.327	0.315	0.318	0.315	−
50%tile	0.453	0.431	0.404	0.386	0.373	−
75%tile	0.610	0.589	0.577	0.553	0.565	−
Max	0.759	0.731	0.700	0.689	0.706	−
Number of designated cancer care hospitals per 10,000 people [29–35]						
Min	−	−	0.000	0.013	0.018	0.019
25%tile	−	−	0.009	0.030	0.033	0.035
50%tile	−	−	0.014	0.038	0.043	0.045
75%tile	−	−	0.028	0.048	0.053	0.056
Max	−	−	0.081	0.084	0.086	0.105
Total population 1000 people [35]						
Min	619	617	610	592	579	552
25%tile	1188	1185	1171	1148	1128	1057
50%tile	1795	1783	1760	1714	1680	1605
75%tile	2871	2872	2871	2853	2863	2789
Max	11500	11800	12300	12700	13400	13800
Percentage of the population aged 65 years and older [35]						
Min	10.3	13.2	15.9	16.9	19.4	22.8
25%tile	15.0	17.5	19.9	22.4	26.2	29.3
50%tile	17.6	19.9	22.5	24.5	28.1	31.0
75%tile	18.7	21.6	23.9	26.1	29.6	33.1
Max	22.0	25.2	27.1	29.1	33.3	37.8
Average income per capita, 1000 yen [36]						
Min	2066	2097	2025	2007	2344	−
25%tile	2633	2496	2484	2413	2632	−
50%tile	2947	2768	2791	2657	2909	−
75%tile	3199	2952	2977	2816	3060	−
Max	4359	4462	5970	5220	5759	−

1 US$ = 149.17 yen (2023/10/12)

SD: Standard Deviation, %tile: percentile

**Fig. 2:**
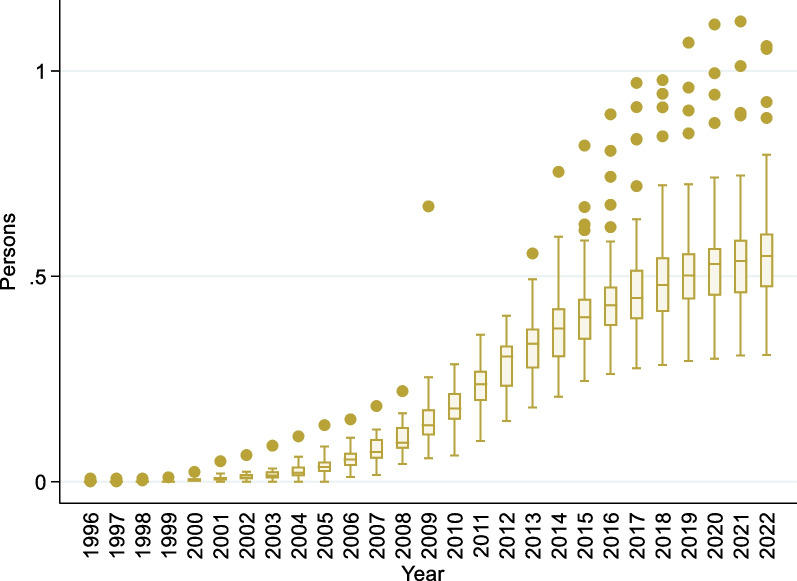
Trends in the number of persons in the APNW in cancer care (per 10,000 people). APNW: Advanced practice nursing workforce

Figure 2 shows the number of persons in the APNW in cancer care per 10,000 people in each prefecture, and a consistent upward trend across all prefectures can be seen until 2009. However, since 2012, when the maximum increase was seen, the minimum remained relatively stable, and the interquartile range (IQR) increased.

**Fig. 3:**
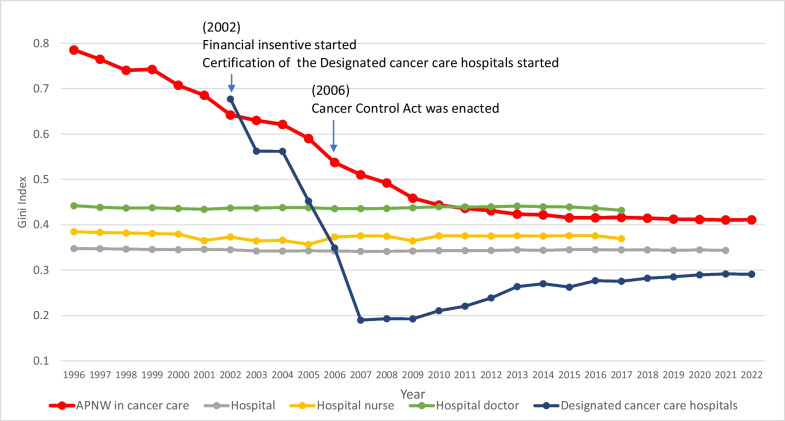
Trends in the Gini index of cancer care resources. APNW: Advanced practice nursing workforce

As shown in Fig. 3, the Gini coefficients for the APNW in cancer care for the total population decreased from 0.79 in 1996 to 0.43 in 2012. However, from 2012 to 2022, the coefficient decreased slightly from 0.43 to 0.41. In the sensitivity analysis, the Gini coefficients for the APNW in cancer care for cancer patients from 2016 to 2019 were 0.424, 0.424, 0.422, and 0.420, respectively. As of 2022, the Gini coefficients for other healthcare resources, including hospitals, hospital doctors, hospital nurses, and designated cancer care hospitals, were 0.34, 0.43, 0.37, and 0.29, respectively.

Finally, a panel data analysis was performed to identify factors related to the size of the APNW in cancer care in each prefecture (Table 2). Year [coefficient: 0.36, 95% confidence interval (CI) 0.33–0.39], more designated cancer care hospitals in the previous year (see first quartile, the coefficient for second quartile: 0.31, 95% CI 0.21–0.40), a lower proportion of older adults in the previous year (− 0.27, 95% CI − 0.32 to − 0.22), lower average income per capita in the previous year (− 1.91, 95% CI − 2.50 to − 1.32) and fewer hospital doctors in the previous year (− 1.89, 95% CI − 2.70 to − 1.09) emerged as factors significantly associated with the APNW in cancer care in each prefecture.

**Table 2:** Fixed-effect panel data regression model for the advanced practice nursing workforces (log) in cancer care

	Univariate regression				Multiple regression											
					Model 1^a^				Model 2^b^				Model 3^c^			
	Coefficient	*P*-value	95%CI		Coefficient	*P*-value	95%CI		Coefficient	*P*-value	95%CI		Coefficient	*P*-value	95%CI	
Number of hospital nurses in the previous year (log)																
	6.998		6.585	7.411	0.632	0.002	0.23	1.034	–	–	–	–	–	–	–	–
Number of hospital doctors in the previous year (log)																
	14.611	< 0.001	13.975	15.246	–	–	–	–	− 1.893	< 0.001	− 2.695	− 1.091	–	–	–	–
Number of hospitals in the previous year (log)																
					–	–	–	–	–	–	–	–	− 0.854	0.098	− 1.865	0.158
Nurses’ wages in the previous year (log)																
	3.087	< 0.001	1.699	4.476	− 0.428	0.128	− 0.979	0.123	− 0.466	0.094	− 1.012	0.08	− 0.913	0.002	− 1.477	− 0.350
Number of designated cancer care hospitals in the previous year																
1st quartile	Reference				Reference				Reference				Reference			
2nd quartile	1.671	< 0.001	1.505	1.836	0.306	< 0.001	0.208	0.404	0.282	0	0.184	0.381	0.369	< 0.001	0.259	0.478
3rd quartile	2.286	< 0.001	2.112	2.461	0.381	< 0.001	0.265	0.496	0.341	0	0.226	0.456	0.659	< 0.001	0.526	0.793
4th quartile	2.853	< 0.001	2.654	3.052	0.429	< 0.001	0.286	0.573	0.445	0	0.303	0.587	0.638	< 0.001	0.486	0.789
Year																
	0.214	< 0.001	0.209	0.22	0.358	< 0.001	0.326	0.39	0.398	0	0.366	0.429	0.333	< 0.001	0.303	0.363
Population in the previous year																
1st quartile	Reference				Reference				Reference				Reference			
2nd quartile	− 2.142	< 0.001	− 2.783	− 1.502	− 0.113	0.289	− 0.322	0.096	− 0.031	0.77	− 0.241	0.179	− 0.066	0.510	− 0.264	0.131
3rd quartile	− 4.668	< 0.001	− 6.074	− 3.262	0.416	0.054	− 0.007	0.839	0.451	0.035	0.031	0.871	0.419	0.051	− 0.001	0.839
4th quartile	− 5.99	< 0.001	− 7.980	− 4.000	0.147	0.668	− 0.525	0.819	0.234	0.491	− 0.433	0.902	0.581	0.059	− 0.022	1.183
Percentage of the population aged 65 + in the previous year																
	0.361	< 0.001	0.35	0.372	− 0.27	< 0.001	− 0.323	− 0.217	− 0.252	< 0.001	− 0.305	− 0.2	− 0.225	< 0.001	− 0.273	− 0.177
Average income per capita in the previous year (log)																
	4.691	< 0.001	3.116	6.267	− 1.91	< 0.001	− 2.503	− 1.318	− 2.456	< 0.001	− 2.998	− 1.915	− 2.730	< 0.001	− 3.263	− 2.196

CI: Confidence interval

^a^Overall *R*^2^ = 0.3681

^b^Overall *R*^2^ = 0.3019

^c^Overall *R*^2^ = 0.4348

## Discussion

To the best of our knowledge, this panel data study is the first to evaluate a 26-year trend in geographic inequity in the APNW size in cancer care at a subnational level in Japan since the CNS and CN certification system was introduced. Further, we identified factors associated with the APNW's size in cancer care.

### The trend in the geographic disparities of the APNW in cancer care in each prefecture in Japan from 1996 to 2022

The number of APNW in cancer care, both the national total and the number per population per prefecture, has been on a consistent upward trend for the past 27 years. This might be explained by an increase in the growth of the nationwide total number of training facilities [39], and financial incentives in the fee schedule [25]. On the other hand, we found that the degree of disparity in the APNW in cancer care decreased from 1996 to 2012 but began increasing from 2012 onwards. Consistent with previous studies on hospital nurses [18] or radiation oncologists [40], we found that despite an increase in their absolute numbers from 2012 to 2022, the degree of disparity remained constant. Studies on the distribution of radiation oncologists in the United States [40] and nurses in Japan [18] have reported persistent imbalances despite increasing numbers. This suggests that merely increasing the APNW in cancer care does not resolve regional disparities.

The narrowing of the gap approximately 15 years after its inception and subsequent stagnation may be explained by two factors. First is the impact of the policy. The multivariable analysis showed a strong association between the number of designated cancer care hospitals and the number of members in the APNW for cancer care. While the distribution of nurses in Japan is influenced by policy [16–18], the distribution of the APNW may also be influenced by policies related to the establishment of designated cancer care hospitals and increased compensation for their placement. Reimbursement based on CNS/CN assignment and medical performance until 2014 may have contributed to the decrease in the Gini coefficient for APNW in cancer care until approximately 2012. However, the lack of new reimbursement policies in the last decade, as well as the increasing specialisation within cancer-designated hospitals, may have contributed to the stagnation of the Gini coefficient after 2012. Moreover, the APNW's size in cancer care may be declining. While the numbers were consistent from 1996 to 2010, they have decreased markedly since 2010, potentially leading to stagnation in the disparity. However, there is limited research investigating the uneven distribution of the nursing workforce in Japan and its correlation with policy influences. Policy evaluations and societal experiments aimed at addressing these disparities will be essential for future initiatives.

### Factors associated with the number of APNW in cancer care

A higher number of persons in the APNW in cancer care is associated with fewer hospital doctors and more designated cancer care hospitals in the previous year after adjusting for the socioeconomic status of the region.

The results that a number of doctors have a negative association with the APNW consist in previous studies for other clinical fields in APNW in the US [41, 42] and Canada [43], suggesting that APNW may substitute for the doctor workforce. A study report has highlighted that advanced practice nurses contributed to alleviating the physicians’ workload by managing the patient's symptoms by substituting for the physicians [7]. In Japan, a nursing training system for specific acts was introduced in 2015, allowing CNs trained under this program to conditionally perform medical acts typically performed by doctors, such as drug administration and managing circulatory dynamics [44]. Thus, there is potential for task shifting from doctors to advanced practice nurses for specific acts.

A larger APNW is associated with a higher number of designated cancer care hospitals, consistent with previous studies showing that the distribution of nurses in Japan is associated with the nurse staffing policy under the low or fee schedule [16–18]. As designated cancer care hospitals are policy-driven in Japan and mandate at least one CNS or CN placement [24], it is plausible that the distribution of nurses is influenced by policy. Future study is necessary to examine the effect of the introduction of the APNW staffing mandate or incentive in the fee schedule on the geographic distribution of APNW.

In this study, the number of hospital nurses was not a statistically significant association, but the point estimate shows a negative association. To be certificated as CN and CNS, a minimum of 600 h of training and 2 years of master’s course education are needed. During the additional training, they have to leave the clinical site [22, 23]. In areas with few hospital nurses, securing enough replacement personnel to make the career advancement to advanced practice nursing providers may prove challenging [45]. In contrast with studies that have highlighted a correlation between the distribution of nurses in Japan and their wages, this study did not find a correlation between wages and the APNW size [17]. Financial incentives have motivated the development of advanced nurses in other countries [46, 47]. A 2022 survey revealed that only in 7.7% of the cases, evaluation and treatment were based on the qualifications of the APNW, while in 87% of the cases, no specific financial rewards were offered based on qualifications [48]. This lack of qualification-based economic incentives might explain the lack of correlation between the size of the APNW and wages. However, Japan's nursing wage elasticity is at 0.1, suggesting that a sudden increase in wages might not directly lead to a considerable increase in the number of nurses [17]. Further research is required to ascertain whether an increase in wages would lead to an increase in the APNW size in Japan.

### Limitations

This study has some limitations. First, comprehensive data on the training institutions were lacking. Previous studies have shown a strong correlation between the distribution of highly specialized nurses, such as CNSs, and training institutions [17, 18, 49]. While the present study could not definitively establish this relationship in oncology care owing to the absence of longitudinal data on their nationwide distribution, it is speculated that there is a certain degree of correlation. Investigating the distribution and changes in training institutions is crucial for further exploring factors related to the distribution of the APNW in cancer care.

Second, insufficient data on the number of cancer patients may not provide an accurate picture of the cancer workforce. To understand the geographical distribution of the healthcare workforce associated with cancer care, a workforce count specific to cancer patients may be more appropriate than the number of healthcare workers per population [3]. In this study, data on the national distribution of cancer patients were available only from 2016 to 2019. Therefore, we used the number of individuals in the APNW in cancer care relative to the total population as a proxy for the demand of the cancer workforce. The sensitivity analysis shows that the Gini coefficients for the APNW in cancer care using the available cancer patient data were similar to those based on the number of cancer patients.

Third, our analyses were based on the prefectural-level CNSs/CNs data. However, disparities among the prefectures were also highlighted [50]. Therefore, it is necessary to examine the distribution of the workforce in smaller units, such as secondary medical care areas, to understand the disparities within prefectures comprehensively.

Fourth, the number of workforce in this study might underestimate the actual nursing service delivery for patients. This study used a headcount number of nurses. And there are 87.2% for CNS and for 94.8% of CNS work clinical settings such as hospitals, long-term care facilities and home visiting nurse agencies (others work nursing university or college or leaving their job) as of December 2023 [22, 23]. Moreover, the APNW certification system by the JNA allows one nurse to have more than two categories of clinical field. For instance, there are possible that one nurse has both certifications CNS in Cancer Nursing and cancer chemotherapy CNs.

## Conclusion

The absolute number of individuals in the APNW in cancer care has increased since its inception. However, although the disparities in APNW in cancer care diminished for approximately 15 years after their establishment, they subsequently plateaued. Additionally, the number of APNW members in cancer care seems to be associated with the number of hospital nurses, the number of designated cancer care hospitals, and the scarcity of hospital doctors.

### Supplementary Information

Supplementary Material 1. Appendix 1 Consolidation process for certified nurses (CNs) in cancer care.

Supplementary Material 2. Appendix 2 Detail information on the advanced practice nursing workforce and prefecture characteristics from 1996 to 2022.

## Data Availability

The datasets generated and analysed during the current study are available in the Japanese Nursing Association repository (https://www.nurse.or.jp/nursing/qualification/vision/cns/index.html and https://www.nurse.or.jp/nursing/qualification/vision/cn/index.html, in Japanese), and Japanese government repository (https://www.e-stat.go.jp/en).

## Abbreviations

CNS: Certified nurse specialist
CN: Certified nurse
APNW: Advanced practice nursing workforce
SD: Standard deviation
%tile: Percentile
IQR: Interquartile range
CI: Confidence interval
JNA: Japanese Nursing Association
