# Supplementary material for: Trends in the geographic inequality of advanced practice nursing workforce in cancer care in Japan from 1996 to 2022: a panel data analysis
Source: Hum Resour Health. 2024 May 27;22:33. doi: 10.1186/s12960-024-00922-z (PMC11131239; doi:10.1186/s12960-024-00922-z)
Supplement: Supplementary file 1 — Supplementary Material 1. Appendix 1 Consolidation process for certified nurses (CNs) in cancer care. [file 12960_2024_922_MOESM1_ESM.pptx]

## Slide 1
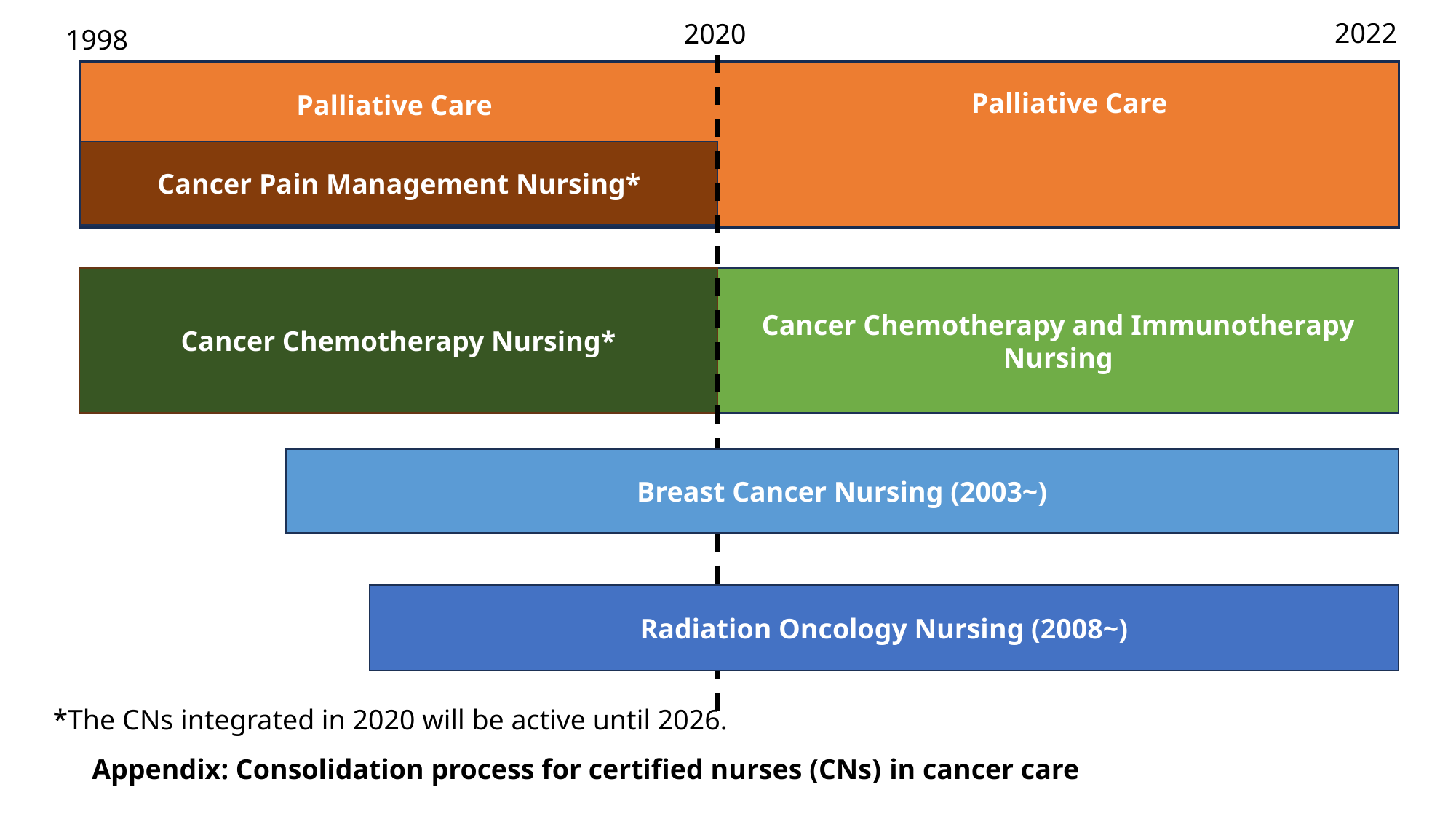

2022
2020
1998
Cancer Pain Management Nursing*
Palliative Care
Palliative Care
Cancer Chemotherapy Nursing*
Cancer Chemotherapy and Immunotherapy Nursing
Breast Cancer Nursing (2003~)
Radiation Oncology Nursing (2008~)
*The CNs integrated in 2020 will be active until 2026.
Appendix: Consolidation process for certified nurses (CNs) in cancer care
