# Supplementary material for: Trends in the geographic inequality of advanced practice nursing workforce in cancer care in Japan from 1996 to 2022: a panel data analysis
Source: Hum Resour Health. 2024 May 27;22:33. doi: 10.1186/s12960-024-00922-z (PMC11131239; doi:10.1186/s12960-024-00922-z)
Supplement: Supplementary file 2 — Supplementary Material 2. Appendix 2 Detail information on the advanced practice nursing workforce and prefecture characteristics from 1996 to 2022. [file 12960_2024_922_MOESM2_ESM.pdf]

Appendix 2. Detailed information on the advanced practice nursing workforce and prefecture characteristics from 1996 to 2022.

Prefecture	Year	Advance practice nursing workforce in cancer care (persons)	Total population (persons)	Percentage of aged 65+	Average income per capita (thousand yen)	Average hourly of salary of nurse (thousand yen)	Number of hospital nurse (persons)	Number of hospital doctor (persons)	Number of hospital	Number of designated cancer care hospitals
Hokkaido	1996	0	5684842	15.1	2835	-	25514	7553	654	0
Hokkaido	2001	4	5675309	18.4	2689	2.42	46103	8093	638	0
Hokkaido	2006	38	5629970	21.5	2528	2.24	33781	8569	613	8
Hokkaido	2011	179	5498916	24.6	2456	2.23	39913	9111	579	20
Hokkaido	2016	266	5401210	28.8	2693	2.47	44815	9745	562	22
Hokkaido	2022	356	5183687	32.3	0	-	0	0	0	22
Aomori	1996	0	1508720	16.1	2536	-	6170	1520	112	0
Aomori	2001	0	1497036	19.6	2329	2.14	9277	1549	110	0
Aomori	2006	5	1460144	22.5	2381	1.89	7224	1638	109	2
Aomori	2011	30	1395886	25.4	2401	2.21	8218	1754	102	5
Aomori	2016	50	1338465	29.6	2544	2.10	8912	1843	96	6
Aomori	2022	70	1243081	33.9	0	-	0	0	0	5
Iwate	1996	0	1430118	18.2	2607	-	6963	1756	110	0
Iwate	2001	1	1421796	21.7	2414	2.51	9733	1814	109	0
Iwate	2006	5	1388164	24.6	2371	2.30	7881	1859	103	1
Iwate	2011	28	1334814	26.8	2413	2.15	8304	1914	92	9
Iwate	2016	69	1289470	30.2	2656	2.10	8727	1988	93	10
Iwate	2022	93	1206479	33.9	0	-	0	0	0	10
Miyagi	1996	0	2311572	15.0	2846	-	7412	2609	152	0
Miyagi	2001	0	2347166	17.7	2646	2.45	12295	2734	149	0
Miyagi	2006	14	2344569	20.2	2648	2.28	10344	2921	149	7
Miyagi	2011	41	2318956	22.2	2494	2.30	11988	3174	141	7
Miyagi	2016	80	2324466	25.3	2987	2.35	13659	3498	139	7
Miyagi	2022	108	2268355	28.7	0	-	0	0	0	8
Akita	1996	0	1222018	20.0	2633	-	5176	1428	86	0
Akita	2001	2	1197566	23.8	2427	2.28	8111	1530	81	0
Akita	2006	7	1156356	26.8	2396	2.35	6411	1563	78	0
Akita	2011	20	1097588	29.1	2326	2.18	7191	1651	75	8
Akita	2016	47	1043015	33.3	2533	2.45	7607	1691	69	9
Akita	2022	66	956836	37.8	0	-	0	0	0	10
Yamagata	1996	0	1254588	20.3	2647	-	5306	1302	69	0
Yamagata	2001	0	1241364	23.3	2407	2.28	7680	1469	69	0
Yamagata	2006	2	1212163	25.7	2556	2.05	6663	1532	70	4
Yamagata	2011	22	1168752	27.3	2436	2.09	7415	1678	68	6
Yamagata	2016	45	1129560	30.6	2719	2.36	7963	1720	68	6
Yamagata	2022	61	1056682	34.2	0	-	0	0	0	6
Fukushima	1996	0	2137990	17.7	2957	-	7819	2375	165	0
Fukushima	2001	0	2133396	20.5	2767	2.36	13804	2380	156	0
Fukushima	2006	6	2100851	22.7	2779	2.07	9355	2441	147	2
Fukushima	2011	31	2036146	24.5	2360	2.15	10233	2473	130	8
Fukushima	2016	64	1953699	28.1	2922	2.07	11145	2646	128	9
Fukushima	2022	77	1841244	32.0	0	-	0	0	0	6
Ibaraki	1996	0	2964839	14.4	3117	-	7423	2956	225	0
Ibaraki	2001	6	2995583	16.8	2895	2.24	14234	3259	209	0
Ibaraki	2006	17	2988533	19.5	2834	2.27	10359	3384	201	4
Ibaraki	2011	41	2973174	22.4	2910	2.37	12565	3649	183	8
Ibaraki	2016	84	2970231	26.2	3111	2.45	14547	4127	178	11
Ibaraki	2022	99	2890377	29.7	0	-	0	0	0	10
Tochigi	1996	0	1982565	15.1	3202	-	5419	2460	119	0
Tochigi	2001	1	2003283	17.5	3003	2.37	10202	2670	118	0
Tochigi	2006	11	2009498	19.6	3230	2.19	7847	2803	115	1
Tochigi	2011	37	1995901	22.0	2964	2.40	9170	3098	109	6
Tochigi	2016	67	1998864	25.7	3338	2.53	10431	3290	107	8
Tochigi	2022	85	1942494	29.3	0	-	0	0	0	8
Gunma	1996	0	2000623	16.0	3077	-	5714	2250	140	0
Gunma	2001	0	2019726	18.5	2853	2.36	11572	2495	144	0
Gunma	2006	13	2020037	20.9	2843	2.31	8527	2591	141	6
Gunma	2011	55	1998558	23.5	2816	2.12	10281	2835	133	9
Gunma	2016	89	2005320	27.1	3249	2.22	12181	3124	129	9
Gunma	2022	109	1943667	30.1	0	-	0	0	0	9
Saitama	1996	0	6718268	10.3	3272	-	13437	5886	371	0
Saitama	2001	2	6898219	13.2	2952	2.39	26848	6374	366	0

Prefecture	Year	Advance practice nursing workforce in cancer care (persons)	Total population (persons)	Percentage of aged 65+	Average income per capita (thousand yen)	Average hourly of salary of nurse (thousand yen)	Number of hospital nurse (persons)	Number of hospital doctor (persons)	Number of hospital	Number of designated cancer care hospitals
Saitama	2006	27	7019919	16.7	2892	2.34	19901	6802	359	4
Saitama	2011	114	7140929	20.4	2793	2.45	25084	7840	346	11
Saitama	2016	188	7323413	24.4	3002	2.38	30619	8850	342	13
Saitama	2022	219	7385848	26.7	0	-	0	0	0	14
Chiba	1996	1	5778793	11.5	3304	-	13951	5587	304	0
Chiba	2001	5	5920398	14.4	2977	2.45	24444	5881	299	0
Chiba	2006	32	6035658	17.7	2977	2.32	19082	6579	286	6
Chiba	2011	114	6161921	21.2	2780	2.62	23952	7535	279	13
Chiba	2016	210	6265899	25.2	3042	2.38	29558	8748	286	14
Chiba	2022	257	6310875	27.5	0	-	0	0	0	15
Tokyo	1996	1	11542468	13.6	4359	-	43456	21053	704	0
Tokyo	2001	16	11818845	16.4	4462	2.65	63459	21544	685	0
Tokyo	2006	100	12273376	18.6	5970	2.70	54610	23103	658	10
Tokyo	2011	340	12662461	20.5	5220	2.67	65359	25913	643	16
Tokyo	2016	590	13415349	22.4	5759	2.67	75541	28202	651	29
Tokyo	2022	726	13794933	22.8	0	-	0	0	0	30
Kanagawa	1996	1	8172001	11.4	3662	-	24134	9235	370	0
Kanagawa	2001	42	8425783	14.2	3279	2.76	36616	9834	363	0
Kanagawa	2006	132	8693373	17.2	3247	2.50	30399	10638	351	7
Kanagawa	2011	285	8906590	20.3	3076	2.74	36293	12272	344	12
Kanagawa	2016	427	9136151	23.7	3119	2.55	42914	13472	341	18
Kanagawa	2022	517	9215210	25.4	0	-	0	0	0	22
Niigata	1996	0	2488917	18.7	2941	-	9377	2589	136	0
Niigata	2001	1	2476900	21.6	2757	2.53	14842	2709	139	0
Niigata	2006	7	2438482	23.9	2715	2.32	11679	2805	140	5
Niigata	2011	34	2378853	26.0	2601	2.46	13445	2907	130	9
Niigata	2016	64	2319435	29.6	2889	2.33	15035	3156	131	9
Niigata	2022	115	2188469	33.0	0	-	0	0	0	9
Toyama	1996	0	1126841	18.1	3428	-	5177	1575	118	0
Toyama	2001	0	1124414	21.0	3083	2.27	8091	1699	115	0
Toyama	2006	4	1114714	23.3	3169	2.27	6516	1713	116	1
Toyama	2011	33	1092885	26.0	2995	2.28	7553	1831	110	8
Toyama	2016	85	1080160	30.0	3129	2.20	8391	1978	106	7
Toyama	2022	108	1037319	32.4	0	-	0	0	0	5
Ishikawa	1996	0	1171986	16.5	3097	-	6069	2034	128	0
Ishikawa	2001	1	1176601	18.9	2989	2.30	9408	2092	118	0
Ishikawa	2006	5	1171106	21.0	2856	2.31	7742	2065	106	1
Ishikawa	2011	24	1160206	23.6	2609	2.17	9000	2229	101	5
Ishikawa	2016	69	1157042	27.5	2947	2.51	9850	2463	95	5
Ishikawa	2022	72	1124501	29.9	0	-	0	0	0	5
Fukui	1996	0	826407	18.0	2961	-	3205	1196	98	0
Fukui	2001	0	828039	20.7	2813	2.26	5672	1278	93	0
Fukui	2006	7	821073	22.5	3336	2.13	4202	1316	83	3
Fukui	2011	27	806428	24.5	2903	2.24	5079	1424	72	5
Fukui	2016	37	799220	28.0	3024	2.31	5744	1480	68	5
Fukui	2022	49	767561	30.6	0	-	0	0	0	5
Yamanashi	1996	0	877794	17.6	3021	-	3002	1042	61	0
Yamanashi	2001	0	886077	19.9	2697	2.42	4946	1097	61	0
Yamanashi	2006	1	879239	22.2	2809	2.20	4061	1218	61	1
Yamanashi	2011	13	860559	24.6	2711	2.29	4510	1286	60	3
Yamanashi	2016	74	849784	27.9	2876	2.33	5075	1411	60	5
Yamanashi	2022	82	816340	31.0	0	-	0	0	0	4
Nagano	1996	0	2190307	19.3	3063	-	8320	2257	145	0
Nagano	2001	3	2204498	21.8	2913	2.40	12933	2602	141	0
Nagano	2006	11	2190874	24.0	2802	2.35	11191	2845	138	3
Nagano	2011	56	2153802	26.4	2616	2.41	12730	3180	133	8
Nagano	2016	100	2137666	29.5	2875	2.41	14509	3428	130	11
Nagano	2022	117	2056970	31.9	0	-	0	0	0	12
Gifu	1996	0	2099352	15.7	2986	-	5890	2089	121	0
Gifu	2001	0	2109804	18.5	2785	2.46	10716	2249	114	0
Gifu	2006	6	2105011	21.3	2822	2.13	8368	2359	108	7
Gifu	2011	40	2076675	24.0	2662	2.32	9794	2571	104	7
Gifu	2016	75	2076195	27.7	2920	2.42	11130	2786	102	7
Gifu	2022	98	1996682	30.4	0	-	0	0	0	8

Prefecture	Year	Advance practice nursing workforce in cancer care (persons)	Total population (persons)	Percentage of aged 65+	Average income per capita (thousand yen)	Average hourly of salary of nurse (thousand yen)	Number of hospital nurse (persons)	Number of hospital doctor (persons)	Number of hospital	Number of designated cancer care hospitals
Shizuoka	1996	0	3734279	15.1	3342	-	11984	3553	183	0
Shizuoka	2001	3	3764054	18.0	3220	2.46	17531	3794	184	0
Shizuoka	2006	17	3775903	20.9	3383	2.48	15278	4225	188	4
Shizuoka	2011	75	3760801	23.7	3073	2.44	17402	4612	186	11
Shizuoka	2016	134	3770619	27.3	3365	2.25	19952	5147	181	12
Shizuoka	2022	182	3658375	30.1	0	-	0	0	0	12
Aichi	1996	0	6770293	12.3	3739	-	21190	8135	394	0
Aichi	2001	5	6935031	15.0	3407	2.45	34559	8298	367	0
Aichi	2006	23	7106585	17.7	3728	2.23	28344	8965	347	5
Aichi	2011	155	7249626	20.5	3273	2.34	33616	9898	327	15
Aichi	2016	272	7509636	23.6	3746	2.45	39240	10718	323	17
Aichi	2022	333	7528519	25.2	0	-	0	0	0	19
Mie	1996	0	1843869	16.3	3034	-	5700	1922	120	0
Mie	2001	0	1858890	19.2	2820	2.48	9927	2076	115	0
Mie	2006	8	1857456	21.7	3166	2.49	7842	2125	112	4
Mie	2011	37	1844293	24.2	2720	2.42	9159	2259	102	6
Mie	2016	68	1850028	27.4	3060	2.44	10496	2603	100	4
Mie	2022	82	1784968	29.9	0	-	0	0	0	5
Shiga	1996	1	1283341	14.4	3450	-	4855	1610	59	0
Shiga	2001	2	1334621	16.4	3202	2.37	7314	1668	60	0
Shiga	2006	8	1365393	18.5	3138	2.30	6958	1899	60	3
Shiga	2011	32	1390927	20.6	2937	2.43	8090	2128	60	6
Shiga	2016	55	1419863	24.0	3226	2.35	9018	2338	57	7
Shiga	2022	61	1415222	26.4	0	-	0	0	0	7
Kyoto	1996	0	2551061	15.3	3059	-	11508	4408	196	0
Kyoto	2001	1	2563205	18.0	2773	2.48	17548	4282	183	0
Kyoto	2006	18	2566420	20.6	2791	2.32	13891	4715	177	1
Kyoto	2011	68	2547225	23.5	2659	2.36	15834	5203	175	9
Kyoto	2016	120	2574842	27.5	2909	2.53	18042	5543	170	12
Kyoto	2022	143	2511494	29.5	0	-	0	0	0	14
Osaka	1996	0	8592991	12.3	3545	-	31365	12747	591	0
Osaka	2001	4	8628601	15.4	3070	2.45	53202	12843	575	0
Osaka	2006	47	8663719	18.8	3241	2.32	40228	13888	549	11
Osaka	2011	201	8681623	22.1	2795	2.21	49065	15562	534	14
Osaka	2016	353	8865502	25.7	2942	2.60	57345	17026	523	17
Osaka	2022	443	8800753	27.1	0	-	0	0	0	18
Hyogo	1996	0	5422446	14.6	3297	-	18785	6527	349	0
Hyogo	2001	4	5537365	17.2	2794	2.36	31338	6791	349	0
Hyogo	2006	49	5576784	19.9	2932	2.13	25458	7203	353	0
Hyogo	2011	139	5580139	22.7	2657	2.39	29925	8315	348	14
Hyogo	2016	219	5621087	26.4	2960	2.50	35085	9536	350	14
Hyogo	2022	255	5488605	28.8	0	-	0	0	0	18
Nara	1996	0	1434579	14.0	3044	-	4432	1634	73	0
Nara	2001	0	1448533	16.8	2784	2.37	7389	1762	74	0
Nara	2006	7	1430366	20.1	2634	2.45	6113	2007	78	1
Nara	2011	40	1406701	23.7	2624	2.51	7420	2231	75	5
Nara	2016	66	1387818	28.3	2669	2.52	8658	2504	77	6
Nara	2022	69	1335378	31.6	0	-	0	0	0	6
Wakayama	1996	0	1098682	18.2	2609	-	3426	1430	95	0
Wakayama	2001	1	1087614	21.2	2508	2.28	6659	1531	92	0
Wakayama	2006	6	1061559	23.8	2776	2.19	5118	1626	94	3
Wakayama	2011	20	1025613	26.4	2728	2.32	6311	1670	92	6
Wakayama	2016	36	994317	30.3	2986	2.37	7003	1887	83	6
Wakayama	2022	46	935084	33.2	0	-	0	0	0	6
Tottori	1996	0	619238	19.5	2679	-	2890	897	42	0
Tottori	2001	0	617078	22.2	2496	2.22	4350	974	46	0
Tottori	2006	3	610434	24.0	2422	2.28	3606	1057	46	2
Tottori	2011	19	592213	25.8	2070	2.16	4110	1088	45	5
Tottori	2016	40	579309	29.3	2359	2.36	4717	1194	44	5
Tottori	2022	48	551806	32.5	0	-	0	0	0	3
Shimane	1996	0	772601	22.0	2514	-	3624	1072	63	0
Shimane	2001	0	762144	25.2	2520	2.13	5568	1180	60	0
Shimane	2006	5	744677	27.1	2484	2.17	4504	1285	60	6
Shimane	2011	22	718218	28.7	2535	2.29	5124	1283	54	5

Prefecture	Year	Advance practice nursing workforce in cancer care (persons)	Total population (persons)	Percentage of aged 65+	Average income per capita (thousand yen)	Average hourly of salary of nurse (thousand yen)	Number of hospital nurse (persons)	Number of hospital doctor (persons)	Number of hospital	Number of designated cancer care hospitals
Shimane	2016	38	701394	32.0	2819	2.28	5648	1322	51	5
Shimane	2022	55	666331	34.4	0	-	0	0	0	5
Okayama	1996	0	1950586	17.7	2926	-	9382	3021	199	0
Okayama	2001	2	1957529	20.4	2753	2.24	14115	3135	192	0
Okayama	2006	8	1954919	22.5	2872	2.10	11884	3372	182	5
Okayama	2011	37	1934057	24.9	2508	2.33	13801	3819	174	7
Okayama	2016	75	1933781	28.3	2773	2.44	15576	4150	164	9
Okayama	2022	78	1879280	30.3	0	-	0	0	0	9
Hiroshima	1996	0	2870671	16.2	3199	-	10829	3673	280	0
Hiroshima	2001	0	2872196	18.8	3035	2.36	19221	3920	270	0
Hiroshima	2006	18	2870907	21.1	3018	2.12	14752	4035	254	10
Hiroshima	2011	99	2852728	23.7	2894	2.34	17738	4386	249	11
Hiroshima	2016	141	2863211	27.3	3211	2.34	20138	4702	244	11
Hiroshima	2022	164	2788687	29.6	0	-	0	0	0	12
Yamaguchi	1996	0	1550419	19.4	2947	-	7059	2028	158	0
Yamaguchi	2001	0	1528944	22.5	2805	2.22	11844	2200	152	0
Yamaguchi	2006	5	1499002	25.1	2916	2.15	8785	2184	150	5
Yamaguchi	2011	37	1455401	27.6	2880	2.30	10452	2318	147	7
Yamaguchi	2016	56	1419781	31.8	3186	2.37	11526	2346	147	8
Yamaguchi	2022	76	1340458	34.7	0	-	0	0	0	8
Tokushima	1996	0	837570	19.0	2758	-	4285	1475	132	0
Tokushima	2001	0	831241	22.0	2768	2.30	6906	1516	131	0
Tokushima	2006	8	816321	24.2	2728	2.24	4853	1467	122	1
Tokushima	2011	18	791242	26.3	2755	2.26	5654	1575	114	4
Tokushima	2016	28	770057	30.3	3043	2.33	6345	1734	112	5
Tokushima	2022	44	726729	33.8	0	-	0	0	0	5
Kagawa	1996	0	1033671	18.5	2937	-	5322	1544	121	0
Kagawa	2001	0	1033248	21.1	2778	2.25	7731	1655	108	0
Kagawa	2006	11	1026088	23.1	2818	2.24	6154	1690	99	3
Kagawa	2011	29	1009794	25.1	2715	2.23	6959	1776	93	5
Kagawa	2016	52	1002173	29.0	2903	2.24	7841	1899	90	5
Kagawa	2022	56	964885	31.5	0	-	0	0	0	5
Ehime	1996	0	1523471	18.7	2809	-	7123	2142	161	0
Ehime	2001	0	1508842	21.6	2560	2.35	11718	2215	156	0
Ehime	2006	10	1486946	23.9	2527	2.09	9193	2214	148	3
Ehime	2011	35	1450262	26.2	2555	1.96	10046	2328	144	7
Ehime	2016	60	1415997	30.1	2632	2.33	11025	2484	141	7
Ehime	2022	78	1341539	33.1	0	-	0	0	0	7
Kouchi	1996	0	825995	20.8	2464	-	4369	1468	150	0
Kouchi	2001	1	817869	23.6	2336	2.25	8539	1614	148	0
Kouchi	2006	4	799121	25.8	2313	2.16	5984	1670	140	2
Kouchi	2011	19	766426	28.3	2261	2.26	6900	1676	137	3
Kouchi	2016	33	740059	32.5	2594	2.26	7784	1819	130	3
Kouchi	2022	39	693369	35.5	0	-	0	0	0	4
Fukuoka	1996	0	4895201	15.1	2885	-	24959	8397	492	0
Fukuoka	2001	2	4979227	17.6	2649	2.26	40140	8434	486	0
Fukuoka	2006	11	5028026	19.9	2686	2.24	31790	9208	475	8
Fukuoka	2011	129	5043494	22.0	2662	1.99	37926	10107	467	15
Fukuoka	2016	241	5122448	25.4	2804	2.23	43325	10847	461	17
Fukuoka	2022	323	5108507	27.9	0	-	0	0	0	24
Saga	1996	0	885599	18.0	2592	-	4072	1253	120	0
Saga	2001	0	882639	20.5	2456	1.92	7175	1358	112	0
Saga	2006	2	872302	22.5	2463	1.93	5301	1399	111	1
Saga	2011	11	855968	24.0	2435	1.97	6358	1554	110	4
Saga	2016	35	842457	27.3	2620	2.04	7190	1737	107	4
Saga	2022	41	812193	30.7	0	-	0	0	0	4
Nagasaki	1996	0	1550220	18.0	2355	-	7369	2450	180	0
Nagasaki	2001	0	1527398	21.0	2217	1.98	12610	2367	176	0
Nagasaki	2006	7	1494879	23.5	2279	2.13	9546	2500	166	4
Nagasaki	2011	44	1440853	25.5	2339	2.13	11205	2684	160	6
Nagasaki	2016	81	1404103	29.2	2586	2.19	12456	2883	151	6
Nagasaki	2022	91	1320055	33.3	0	-	0	0	0	6
Kumamoto	1996	0	1865373	18.6	2416	-	9824	2829	234	0
Kumamoto	2001	3	1870416	21.5	2354	1.99	15915	2906	226	0

Prefecture	Year	Advance practice nursing workforce in cancer care (persons)	Total population (persons)	Percentage of aged 65+	Average income per capita (thousand yen)	Average hourly of salary of nurse (thousand yen)	Number of hospital nurse (persons)	Number of hospital doctor (persons)	Number of hospital	Number of designated cancer care hospitals
Kumamoto	2006	9	1858522	23.7	2284	1.93	12194	3059	219	2
Kumamoto	2011	39	1828471	25.3	2271	1.97	14110	3323	216	8
Kumamoto	2016	81	1810343	28.5	2530	2.13	15837	3537	212	7
Kumamoto	2022	102	1747513	31.6	0	-	0	0	0	7
Ooita	1996	0	1241164	18.9	2695	-	5704	1666	164	0
Ooita	2001	1	1234429	21.9	2642	2.09	9784	1906	163	0
Ooita	2006	7	1221714	24.2	2532	1.89	7624	1970	165	2
Ooita	2011	34	1201901	26.3	2391	1.83	9054	2207	160	6
Ooita	2016	61	1183961	30.0	2565	2.12	9919	2313	157	6
Ooita	2022	78	1131140	33.3	0	-	0	0	0	6
Miyazaki	1996	0	1188070	17.7	2313	-	4925	1590	166	0
Miyazaki	2001	0	1184535	20.9	2237	1.85	9280	1613	153	0
Miyazaki	2006	2	1172402	23.4	2212	1.89	6951	1658	145	4
Miyazaki	2011	17	1147867	25.3	2121	2.02	8267	1759	142	3
Miyazaki	2016	33	1128078	29.1	2392	2.14	8969	1897	140	3
Miyazaki	2022	43	1078313	32.8	0	-	0	0	0	3
Kagoshima	1996	0	1794951	20.2	2318	-	7801	2618	292	0
Kagoshima	2001	2	1783231	23.0	2294	1.88	15750	2710	289	0
Kagoshima	2006	9	1759650	24.9	2243	1.86	10774	2855	277	2
Kagoshima	2011	41	1713984	26.1	2242	1.80	13054	3003	265	7
Kagoshima	2016	86	1679502	29.0	2446	2.01	14670	3234	252	12
Kagoshima	2022	105	1605419	32.6	0	-	0	0	0	12
Okinawa	1996	0	1287023	11.6	2066	-	5086	1548	88	0
Okinawa	2001	1	1334122	13.9	2097	1.95	9179	1669	95	0
Okinawa	2006	2	1381820	15.9	2025	2.05	7280	2174	94	2
Okinawa	2011	14	1413583	16.9	2007	2.37	8970	2412	95	3
Okinawa	2016	40	1461231	19.4	2344	1.98	10728	2708	94	6
Okinawa	2022	90	1485670	22.9	0	-	0	0	0	6
